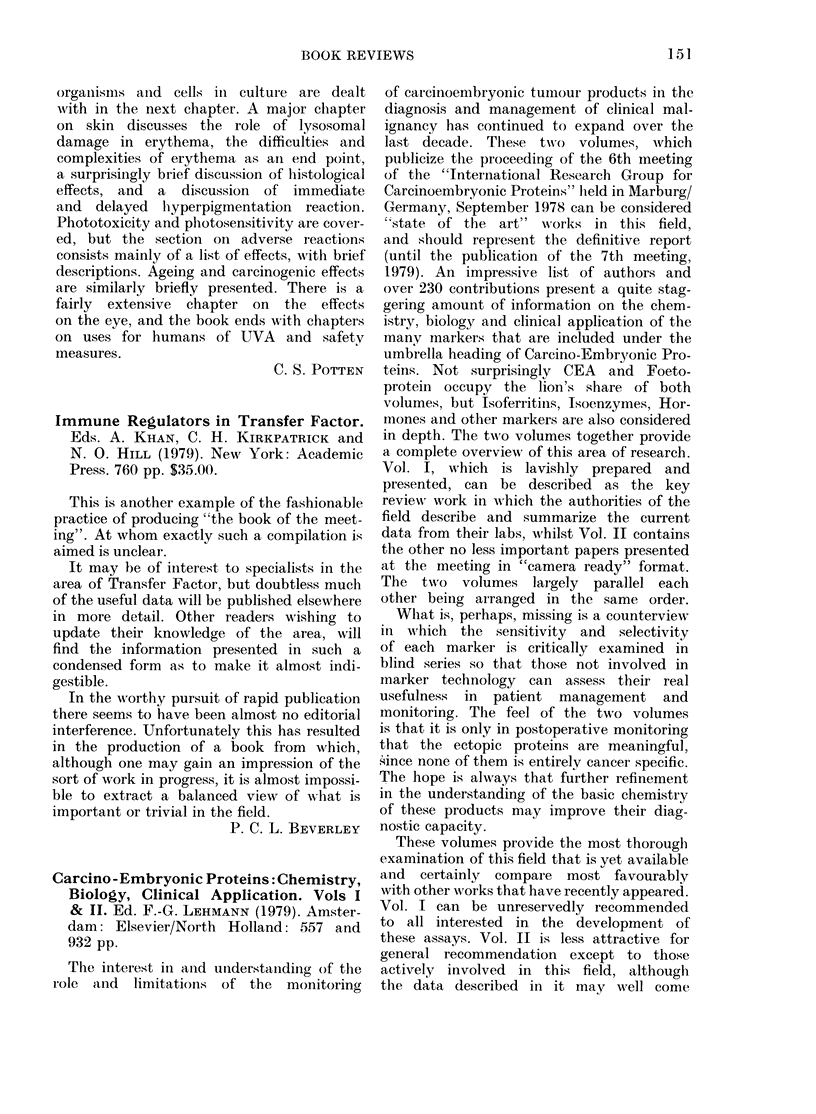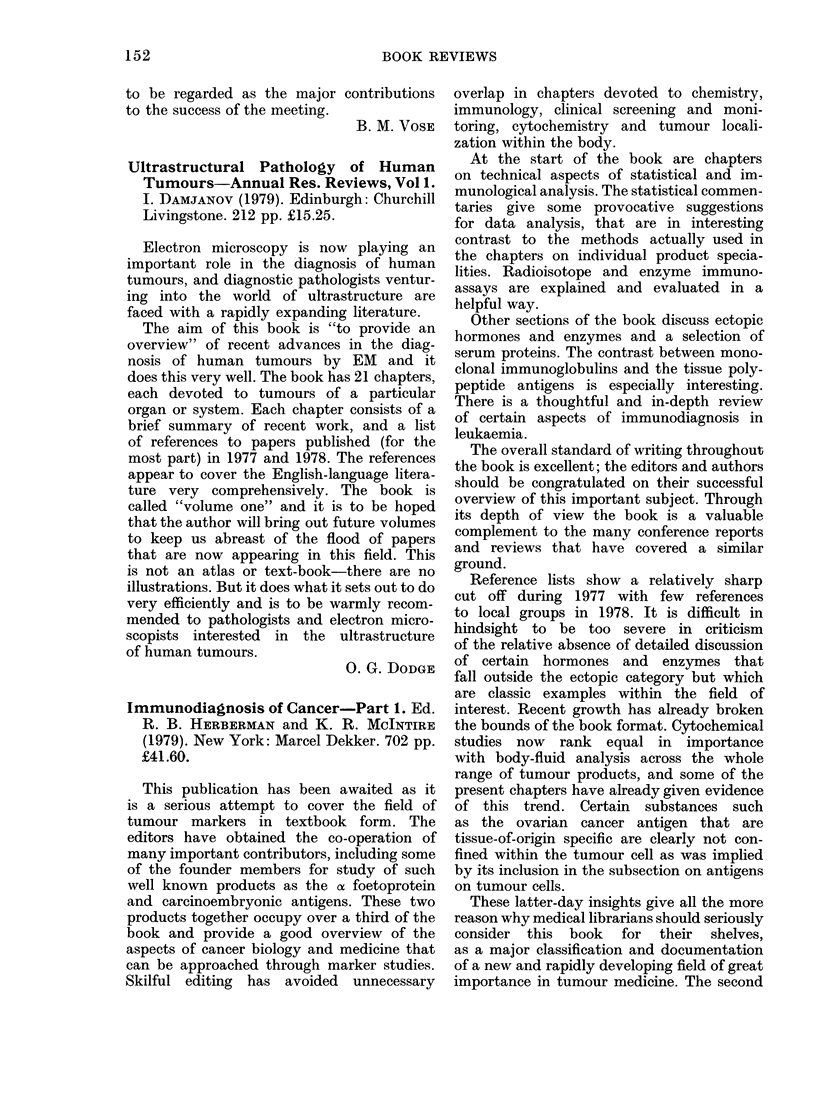# Carcino-Embryonic Proteins: Chemistry, Biology, Clinical Application. Vols I & II

**Published:** 1980-01

**Authors:** B. M. Vose


**Carcino-Embryonic Proteins: Chemistry, Biology, Clinical Application. Vols I & II.** Ed. F.-G. LEHMANN (1979). Amsterdam: Elsevier/North Holland: 557 and 932 pp.

The interest in and understanding of the role and limitations of the monitoring of carcinoembryonic tumour products in the diagnosis and management of clinical malignancy has continued to expand over the last decade. These two volumes, which publicize the proceeding of the 6th meeting of the "International Research Group for Carcinoembryonic Proteins" held in Marburg/Germany, September 1978 can be considered "state of the art" works in this field, and should represent the definitive report (until the publication of the 7th meeting, 1979). An impressive list of authors and over 230 contributions present a quite staggering amount of information on the chemistry, biology and clinical application of the many markers that are included under the umbrella heading of Carcino-Embryonic Proteins. Not surprisingly CEA and Foetoprotein occupy the lion's share of both volumes, but Isoferritins, Isoenzymes, Hormones and other markers are also considered in depth. The two volumes together provide a complete overview of this area of research. Vol. I, which is lavishly prepared and presented, can be described as the key review work in which the authorities of the field describe and summarize the current data from their labs, whilst Vol. II contains the other no less important papers presented at the meeting in "camera ready" format. The two volumes largely parallel each other being arranged in the same order.

What is, perhaps, missing is a counterview in which the sensitivity and selectivity of each marker is critically examined in blind series so that those not involved in marker technology can assess their real usefulness in patient management and monitoring. The feel of the two volumes is that it is only in postoperative monitoring that the ectopic proteins are meaningful, since none of them is entirely cancer specific. The hope is always that further refinement in the understanding of the basic chemistry of these products may improve their diagnostic capacity.

These volumes provide the most thorough examination of this field that is yet available and certainly compare most favourably with other works that have recently appeared. Vol. I can be unreservedly recommended to all interested in the development of these assays. Vol. II is less attractive for general recommendation except to those actively involved in this field, although the data described in it may well come to be regarded as the major contributions to the success of the meeting.

B. M. VOSE